# Dosimetric evaluation of VMAT and helical tomotherapy techniques comparing conventional volumes with clinical target volumes based on new ESTRO ACROP post-mastectomy with immediate implant reconstruction contouring guidelines

**DOI:** 10.1186/s13014-022-02134-y

**Published:** 2022-10-21

**Authors:** Evren Ozan Göksel, Evrim Tezcanli, Alptekin Arifoğlu, Halil Küçücük, Öznur Şenkesen, Ufuk Abacıoğlu, Işık Aslay, Meriç Şengöz

**Affiliations:** 1 Radiotherapy Program, Vocational School of Health Services, Acibadem MAA University, Istanbul, Turkey; 2 General Senology Department, Research Institute of Senology, Acibadem MAA University, Istanbul, Turkey; 3 Department of Radiation Oncology, Acibadem Altunizade Hospital, 34662 Uskudar, Istanbul, Turkey; 4 Department of Radiation Oncology, Acibadem MAA University, Istanbul, Turkey

**Keywords:** VMAT, Helical tomotherapy, ESTRO-ACROP guideline, Post-mastectomy radiation therapy, Implant-based immediate breast reconstruction

## Abstract

**Background:**

The ESTRO-ACROP Consensus Guideline (EACG) recommends implant excluded clinical target volume (CTVp) definitions for post-mastectomy radiation therapy after implant-based immediate breast reconstruction (IBR). The purpose of this study is to investigate the effectiveness of Helical Tomotherapy (HTp) and Volumetric Modulated Arc Therapy (VMATp) treatment techniques in terms of CTVp coverage and reduced organ at risk (OAR), normal tissue and implant doses when CTVp was used for treatment planning as the target structure instead of conventional CTV.

**Methods:**

Eight left-sided and eight right-sided breast cancer patients who underwent IBR after mastectomy were included in this study. Planning CT data sets were acquired during free breathing and patients were treated with HT technique targeted to conventional CTV. Retrospectively, CTVp was delineated based on EACG by the same radiation oncologist, and treatment plans with HTp and VMATp techniques were generated based on CTVp. For each patient, relevant dosimetric parameters were obtained from three different treatment plans.

**Results:**

There was no statistically significant difference on target coverage in terms of, PTVp-D95, PTVp-Vpres, homogeneity index (*p* > 0.05) between HTp and VMATp plans. But, the conformity numbers were significantly higher (HTp vs VMATp, 0.69 ± 0.15 vs 0.79 ± 0.12) for VMATp (Z = − 2.17, *p* = 0.030). While HTp significantly lowered Dmax and Dmean for LAD (LAD-D_max_: χ^2^ = 12.25, *p* = 0.002 and LAD-D_mean_: χ^2^ = 12.30, *p* = 0.002), neither HTp nor VMATp could reduce maximum and mean dose to heart (*p* > 0.05). Furthermore, heart volume receiving 5 Gy was significantly higher for VMATp when compared to HTp (21.2 ± 9.8 vs 42.7 ± 24.8, *p*: 0.004). Both techniques succeeded in reducing the mean dose to implant (HTp vs HT, *p* < 0.001; VMATp vs HT, *p* < 0.001; VMATp vs HTp, *p* = 0.005).

**Conclusion:**

Both HTp and VMATp techniques succeeded to obtain conformal and homogeneous dose distributions within CTVp while reducing the mean implant dose. HTp was found to be superior to VMATp with regards to lowering all OAR doses except for CB.

## Introduction

The importance of post-mastectomy radiation therapy (PMRT) has been well established by several milestone studies and PMRT was shown to provide a clinical benefit for patients with positive lymph nodes and high risk disease [1–4].

Although breast conserving management is the current standard of care for many breast cancer patients, mastectomy applications with immediate breast reconstruction (IBR) with permanent implants have been increasingly used for the last decade especially for patients with BRCA 1–2 mutations [5, 6].

The initial strategy for reconstruction was to use a tissue expander during radiotherapy, followed by permanent implant placement several months after the completion of RT. However, more recently there has been a shift to immediate permanent implant reconstruction for breast cancer patients undergoing mastectomy. Although immediate implant reconstruction provides several benefits for the patients, the IBR might cause some challenges for radiation treatment planning. The target volume in patients with implants to undergo RT after mastectomy is traditionally all breast (CTV) plus peripheral lymph nodes, similar to simulator-based irradiation [7, 8]. When the target volume is determined by this conventional method, the circumference of the implant is also exposed to the same high dose as prescribed to the target, which increases the rate of capsular contracture (CC) of the implant that would lead to inferior cosmesis.

Target volume definitions were revisited for PMRT with IBR patients in the European Society of Radiation & Oncology and Advisory Committee on Radiation Oncology Practice (ESTRO-ACROP) Consensus Guidelines (EACG) [9]. Their recommendations for the clinical target volume chest wall (CTVp) are based on the observation that most of the local recurrences after mastectomy occur at the level of the skin and subcutaneous tissue (range 72–100%), where most of the residual glandular tissues and draining lymphatics are found [7, 8]. The guideline evaluates patients in three groups according to the implant placement and recommends different CTVp definitions for each group with limited volumes compared to the traditional target volume since the implant volume is not included within the CTV. While patients with retropectoral implants were grouped as A (retropectoral with full coverage by the pectoral muscle) and B (retro-pectoral with partial coverage by the pectoral muscle and supportive material in the lower part), patients with pre-pectoral implants were grouped as C (pre-pectoral with full coverage by supportive material). This strategy is initially recommended to limit the doses to the implant as well as to organs at risk (OAR). Although the effectiveness and benefits of this treatment are still under investigation, some selected patients can be offered this strategy [10].

The target volume of CTVp especially for group A patients defined according to the guidelines created a more concave and complex shape than conventional CTV and moved away from the OAR on the ipsilateral side. While this new CTVp makes the treatment plan more challenging in terms of providing conformal and homogeneous dose distribution in the target structure, on the contrary, moving the target away from OAR can aid in meeting dose constraints.

The EACG hypothesized that the use of modern volume-based RT planning may reduce the dose to OAR and implant without compromising target coverage. There are several dosimetric studies showing volumetric modulated arc therapy (VMAT) and helical Tomotherapy (HT) dose-volume histogram (DVH) parameters as well as dose homogeneity might be superior to field in field (FinF) technique for PMRT patients [11–15]. However, to our knowledge there are no studies comparing HT and VMAT techniques based on the target volume based on EACG recommendations for IBR patients receiving PMRT.

The aim of this study is to investigate the effectiveness of HTp and VMATp treatment techniques for achieving more homogeneous and conformal dose distribution when highly concave CTVp (EACG GroupA) was used as the target volume. Furthermore, we aimed to evaluate the OAR, normal tissue (NT) and implant doses when CTVp was used for treatment planning as the target structure instead of conventional CTV.

## Materials and method

### Patient characteristics

Sixteen subsequent patients who underwent IBR after mastectomy treated with PMRT between June 2019 and February 2021 were included in this study. All patients had retropectoral implants. Eight patients included in this study had left-sided breast cancer and eight had right-sided breast cancer. Median patient age was 41 years (32–47 years). At initial diagnosis, 9 patients had stage II breast cancer while 7 had stage III.

To eliminate interobserver variations, target volume and OARs were contoured by the same radiation oncologist and treatment plans were created by the same medical physicist. The study was approved by the Ethics committee before the start (Date: 21.04.2021, Registration number: 2021-08/34).

### Patient setup

The patients were positioned on a breast board in a head-first supine position with the ipsilateral arm abducted above the head while the chin was pointing at the contralateral shoulder. CT images were obtained during free breathing for both right and left sided cases using a Siemens Force CT-Simulator with a slice thickness of 3 mm.

### Target volume definition

The treatments were planned and applied with HT technique based on conventional target volumes. The RTOG Breast Contouring Atlas was used for conventional CTV delineation [16]. Total CTV included chest wall plus axilla level 1, 2, 3 and supraclavicular lymph nodes.

To compensate for inter and intra fractional errors, CTV total was expanded by 5 mm to create PTV.

All patients included in our study had retropectorally inserted implants, therefore the clinic target volumes (CTVp) of the patients were retrospectively re-determined according to the Group A as described in the EACG. The total CTVp was created by combining CTVp, axilla level 1, 2, 3 and supra lymph nodes. Then the total CTVp was expanded by 5.0 mm circumferentially to create PTVp. After PTVp was created, re-planning was done using both VMATp and HTp techniques based on this new PTVp.

### Organs at risk delineation

The heart and left anterior descending coronary artery (LAD) were delineated using RTOG recommendations [16]. The ipsilateral lung (IL), contralateral lung (CL), contralateral breast (CB), liver, entire body and implant volumes were delineated. Lung and body contours were automatically outlined and manually corrected.

### Plan objectives

The prescription dose was 50.4 Gy in 28 fractions for both plans optimized to PTV and PTVp. The first objective was that 95% of the target volume should receive the 95% of the prescription dose and the 1% should not exceed 107% of the prescription dose [18, 19]. Dose restrictions for the OAR were determined according to QUANTEC recommendations in HT plans (Table 1), however these doses were reduced to the lowest possible values in HTp and VMATp plans [19]. While the treatment plans optimized to PTVp used constraints to keep the average dose of the implant below 38 Gy, there were no implant dose constraints for plans created for the conventional PTV. Both HTp and VMATp plans aimed to leave the hotspots outside the implant volume. Liver doses were limited only for right-sided breast cancer patients. Although LAD and heart doses were constrained in both right and left sided patients, LAD and heart doses for only left sided patients were evaluated for the purpose of this study.

**Table 1:** Dose-volume objectives for target structures and organs at risk

Target structures	%95 of PTV and PTVp should receive > %95 of prescribed dose and D1 < 107% of prescribed dose
Heart	Dmax < 35 Gy Dmean < 5 Gy
LAD	Dmax < 25 Gy Dmean < 10 Gy
Ipsilateral lung	V20 < %20 V5 < %65
Contralateral lung	Dmax < 25 Gy Dmean < 5 Gy
Contralateral breast	Dmax < 20 Gy Dmean < 5 Gy
Implant	Dmean < 38 Gy No hotspots inside
Implant-PTV	Dmean < 32 Gy
Liver	V20Gy < %1

### HT planning technique

**Fig. 1:**
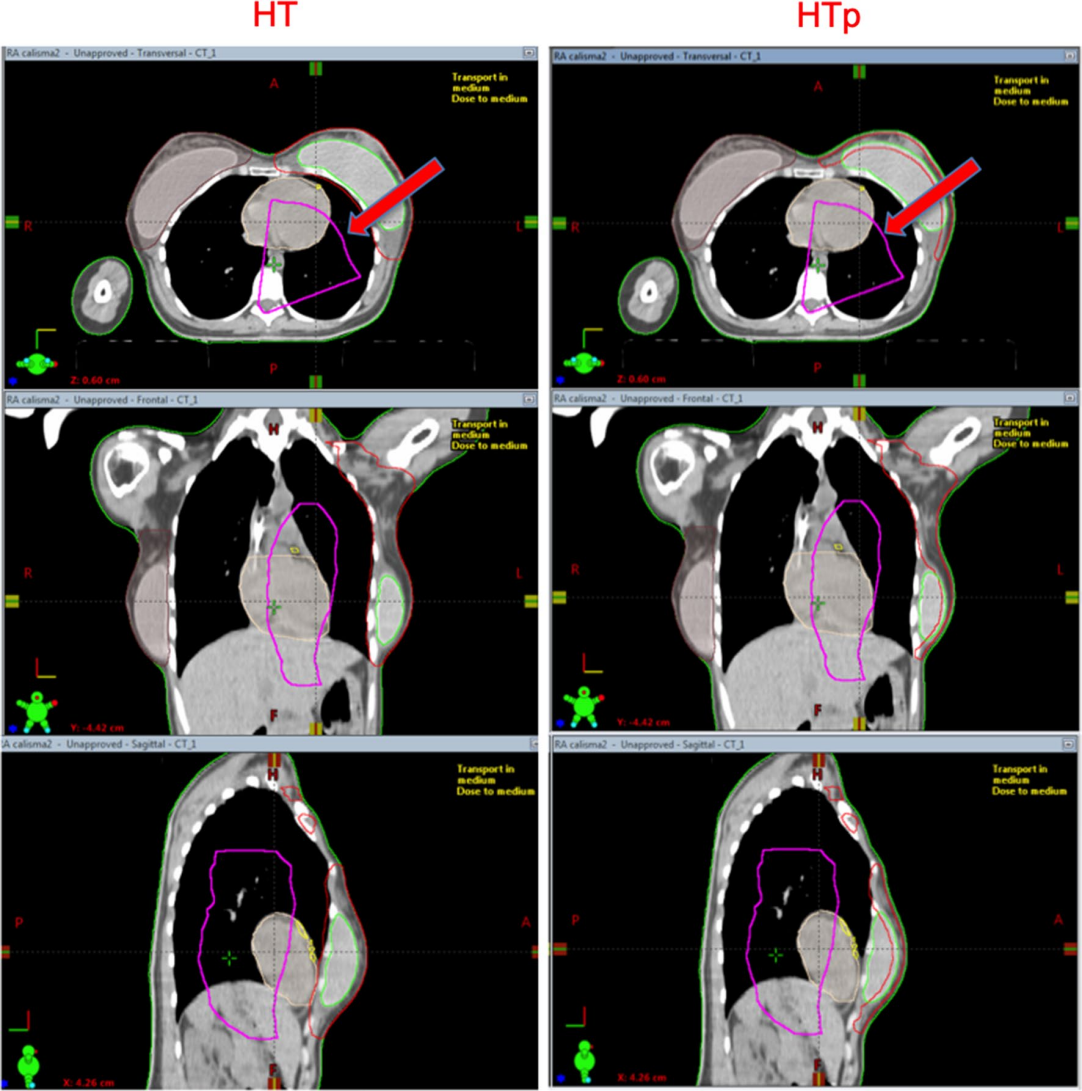
The teardrop shaped dummy structure in HT and HTp plan

HT and HTp plans were generated using the Accuray treatment planning system (TomoHDA Version 2.1.4). The same treatment planning technique was used for both HT and HTp plans. A teardrop-shaped structure was created as a virtual structure during the pre-planning process in order to control OAR doses more easily (Fig. 1). Complete blocking was applied to this dummy structure during the optimization process so that no beams were allowed to enter or exit through heart, LAD, lungs or liver. In addition, four-stage one cm shells were created to control the dose fall off. The field width and pitch was 5.048 cm and 0.287 respectively for both HT and HTp plans. The modulation factor was chosen in the range from 3.5 to 4.0 to achieve conformal dose distributions depending on the anatomy of the patient.

### VMAT planning technique

**Fig. 2:**
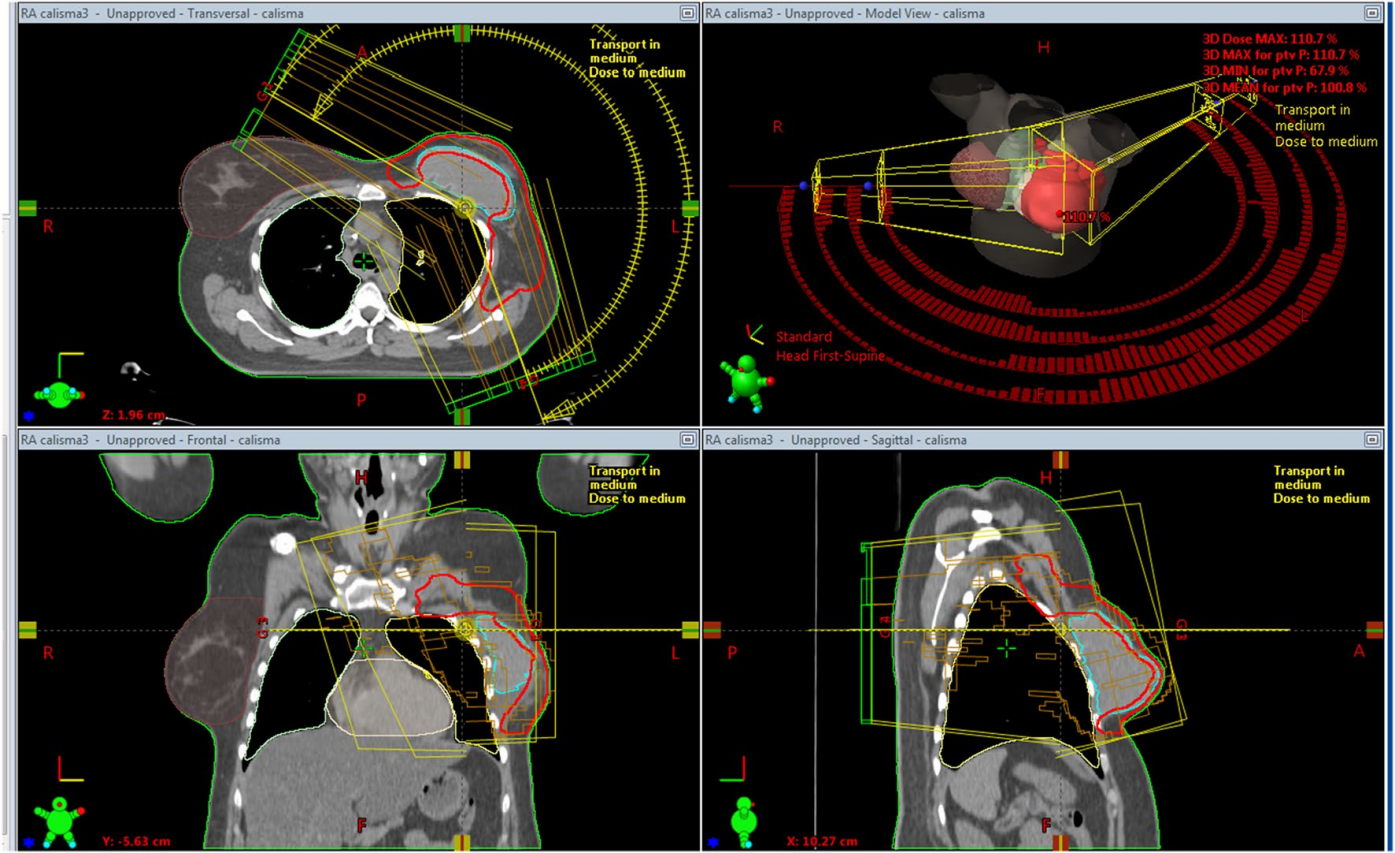
The partial arc arrangement for VMATp plans

For VMATp planning, Varian Eclipse (Version 13.6) treatment-planning system was used and Varian VitalBeam treatment machine equipped with millennium MLC system was chosen. In all VMATp plans, 6MV photon energy and four partial arcs were used (Fig. 2). A partial arc arrangement was selected in order to minimize OAR doses. The starting and ending beam angles of the arcs were determined by using BEV according to the anatomical positions of both PTVp and CB. Collimator angles were adjusted to maximize protection in IL and CB as well as to reduce the tongue-groove effect. The Photon Optimizer algorithm was chosen for VMAT optimization and Acuros XB algorithm was used for dose calculation. The normal tissue objective feature was used to control the dose fall off during the optimization process.

### Plan evaluation

Dose matrices for HT and HTp treatment plans were exported to Eclipse from Accuray treatment planning system for quantitative dosimetric comparison of three different treatment techniques via DVH generation and evaluation of 3D dose distribution. For DVH analysis, Vx was defined as the percentage of a given tissue volume receiving at least x Gy. Dx% was defined as the dose delivered to x% of the volume.

HTp and VMATp plans were compared with HT plans based on DVH parameters and dose distributions for OAR, NT and implant. Another dosimetric comparison was also done between HTp and VMATp plans in order to evaluate the dose homogeneity and conformity for the more concave shaped PTVp when compared with PTV.

ICRU83 homogeneity index (ICRU83-HI) and Conformity number (CN) were used to evaluate dose homogeneity and conformity within PTVp [17, 20]. PTVp-D98 and PTVp-Vpres parameters were also investigated to compare PTVp coverage. The greatest dose to 2% of the volume, D2%, was recorded as an indicator of high dose for the PTVp (Table 2).

**Table 2:** Dosimetric comparison of PTVp coverage (n = 16) (Avg ± STD and *p* values)

Metric	HTp	VMATp	*Z*	*p* value
	Mean(SD)	Mean(SD)		
PTVp-D98	46.90 (8.8)	46.92(7.79)	*−* *0.233*	*0.816*
PTVp-D2	52.46 (5.92)	52.52(9.17)	*−* *0.621*	*0.535*
PTVp-Vpres	45.9(14.1)	53.1(21,02)	*−* *0.569*	*0.569*
PTVp-V95	95.7(0.004)	94.8(0.009)	*0.000*	*0.999*
HI (ICRU83)	0.11(0.02)	0.11(0.02)	*−* *0.724*	*0.469*
CN	0.69(0.15)	0.79(0.12)	***−*** ***2.172***	0.030*

Results with statistical significance are shown in bolditalics

Italics is used for general statistical results

Z, Wilcoxon signed-rank test; Vpres, prescribed dose volume (%) inside of the PTV; Vx, volume (%) receiving x dose (Gy) or higher. Dx%, dose delivered to x% of the volume. SD, standard deviation; CN, conformity number; HI, homogeneity index

*p < 0.05

The detailed OAR doses for all plans are presented in Table 3. Implant Dmax and Dmean doses were evaluated to assess the level of dose reduction using this implant sparing planning strategy. Additionally, body volume receiving 5 Gy was investigated to evaluate the effect of implant sparing planning technique to NT doses.

**Table 3:** Dosimetric comparison of organs at risk dose-volume metrics (n = 16) (Avg ± STD and *p* values)

Metric	HT	HTp	VMATp	*χ*^*2*^	*p* value	Diff.**
	Mean (SD)	Mean (SD)	Mean (SD)			
Heart Dmax (Gy)	30.95 (10.37)	28.32 (7.36)	26.76 (8.73)	*5.375*	*0.068*	–
Heart Dmean (Gy)	4.10 (0.86)	4.57 (1.62)	5.65 (1.86)	*5.375*	*0.068*	–
Heart V5	21.2 (9.8)	30 (20.4)	42.7 (24.8)	***9.125***	0.010*	***c* > *a***
LAD Dmax (Gy)	24.92 (14.59)	9.15 (4.13)	19.49 (5.58)	***12.250***	0.002*	***b* < *a,c***
LAD Dmean (Gy)	8.94 (7.85)	4.25 (1.19)	8.33 (1.27)	***13.000***	0.002*	***b* < *a,c***
Ipsilat Lung V5 (%)	39.2 (4.4)	44.8 (9.4)	91.7 (7.3)	***24.500***	< 0.001*	***c* > *a,b***
Ipsilat Lung V20 (%)	14.3 (1.8)	10.8 (3.1)	18.6 (6.3)	***21.500***	< 0.001*	***b* < *a,c&a* < *c***
Contralat Lung Dmax (Gy)	26.33 (5.36)	23.77 (7.16)	21.56 (5.96)	***10.500***	0.005*	***a* > *c***
Contralat Lung Dmean (Gy)	4.75 (1.36)	5.233 (1.56)	5.485 (1.26)	***10.500***	0.005*	***a* < *c***
Contralat Lung V5 (%)	32.4 (12.2)	35.8 (14.9)	50.1 (18.5)	***10.500***	0.005*	***c* > *a,b***
Contralat breast Dmax (Gy)	22.02 (5.13)	24.28 (3.46)	21.07 (5.19)	*2.625*	*0.269*	–
Contralat breast Dmean (Gy)	5.09 (1.33)	6.17 (1.52)	3.36 (1.46)	***21.125***	< 0.001*	***c* < *a,b***
Implant Dmax (Gy)	53.48 (1.63)	53.79 (1.55)	53.68 (1.01)	*0.875*	*0.646*	–
Implant Dmean (Gy)	50.24 (0.40)	40.88 (2.71)	37.48 (3.33)	***27.125***	< 0.001*	***a* > *b* > *c***
Liver V20 (cc)	0.2 (0.1)	0.8 (0.4)	0.9 (0.5)	*3.714*	*0.156*	–
Normal tissue V5 (cc)	6817.7 (3285.7)	7125.2 (3364.9)	7794.8 (3813.2)	***10.145***	0.006*	***c* > *a***

Results with statistical significance are shown in bolditalics

Italics is used for general statistical results

χ^2^, Friedman test; Dmax, max dose; Dmean, mean dose; Vx,volume (%) receiving x dose (Gy) or higher; SD,Standard deviation; Diff, difference

*p < 0.05; **p < 0.02 (0.05/3) Wilcoxon signed-rank test

### Statistical analysis

Statistical analysis was performed using SPSS (Statistical Package for the Social Sciences) version 25.0 (IBM Corp., Armonk, NY, USA). The normality of the scores obtained from each continuous variable was examined by descriptive, graphical and statistical methods. Shapiro–Wilk test was used to test the normality of the scores obtained from a continuous variable by statistic methods. Nonparametric tests were used for analyses given the normality of distributions and the small sample size (n = 16). In addition to descriptive statistical methods (number, percentage, mean, median, standard deviation, etc.), comparisons between two radiotherapy plans in quantitative data were obtained using the Wilcoxon signed-rank test; comparisons between more than two treatment plans were made with the Friedman test. When comparing more than two groups, Wilcoxon signed-rank test was used to determine which groups caused the difference. Since a maximum of 3 different subgroups were compared, significance was accepted at *p* < 0.02 (0.05/3 = 0.0167) in pairwise comparisons. The results were evaluated within the 95% confidence interval and the significance was evaluated under *p* < 0.05. Accurate sample size was calculated by a method developed by Cohen (d-value) based on the study by Leonardi et al. G-power program (version 3.1) was used to calculate the sample size of 16 with 95% confidence interval (1 − α), 90% power (1 − β) with an estimated mean difference of 4.5 between two groups [21].

## Results

The PTV volume ranged from 972 to 1658 cc (mean, 1281 cc), while PTVp reduced to 485–1085 cc (mean 758 cc). Implant volumes ranged from 292 to 869 cc (mean 458 cc). The differences between HTp and VMATp plans for PTVp-D98, PTVp-D2 and HI parameters were not statistically significant. Although VMATp technique provided slightly better dose distribution with regards to PTVp-Vpres and CN, the difference was only significant for CN (Z = − 2.17, *p* = 0.030). Table 2 compared VMATp and HTp plans with regards to PTVp target coverage. Average values with standard deviations and *p* values were provided.

**Fig. 3:**
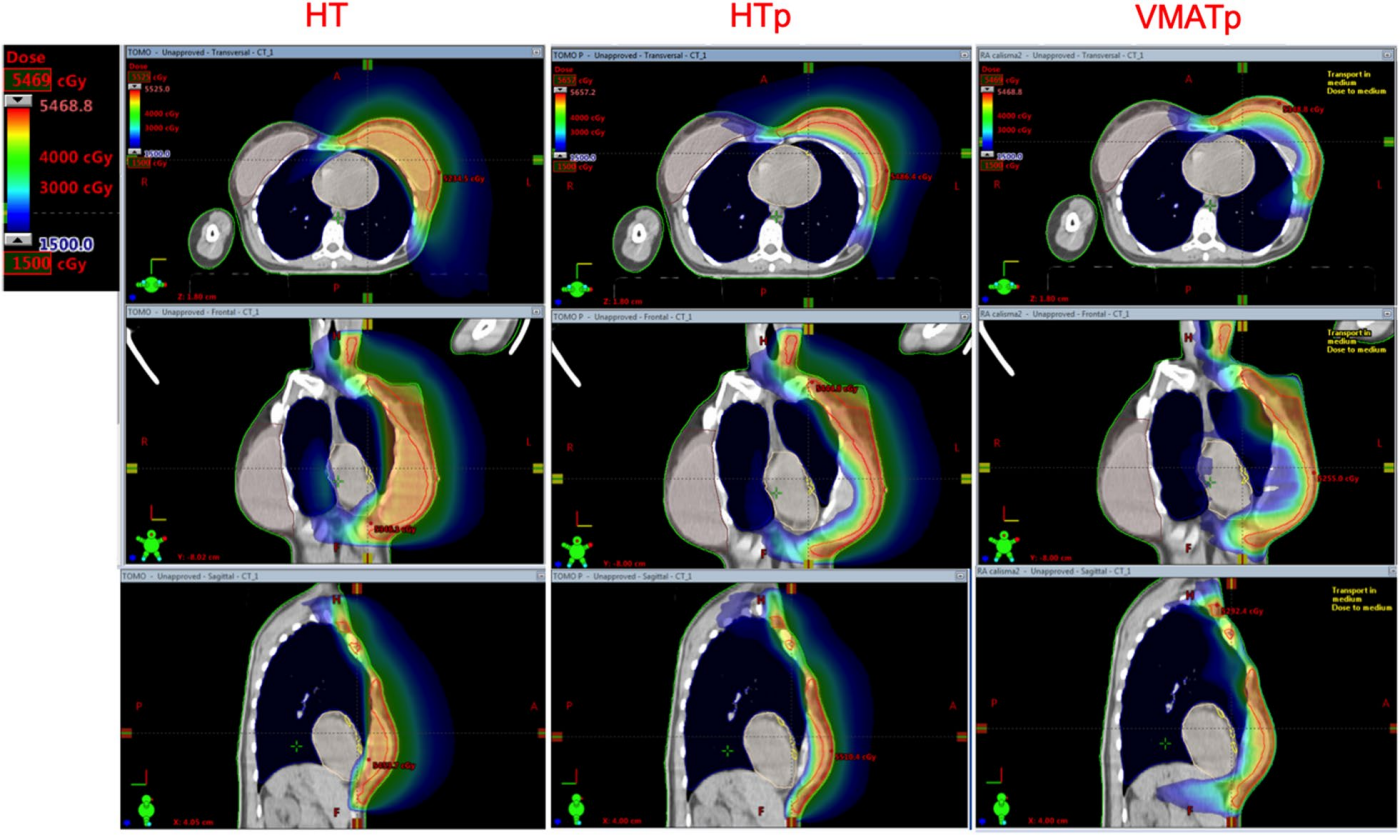
The figure shows representative dose distributions of 15 Gy for HT, HTp and VMATp techniques

HT vs HTp; HT vs VMATp; HTp vs VMATp plan comparisons with regards to dosimetric parameters for OAR, NT and implants were provided with average and *p* values in Table 3. In addition, dose distribution comparisons for all three techniques were presented in Fig. 3.

Target delineation by exclusion of implant from the CTV as per EACG recommendations decreased mean LAD dose (8.94 for HT vs. 4.25 for HTp and 8.33 Gy for VMATp) as well as maximum doses to LAD (24.92 for HT vs 9.15 for HTp and 12.49 Gy for VMATp) and heart (30.95 for HT vs. 28.32 for HTp and 26.76 Gy for VMATp) when compared to conventional contouring that included the implant. However, reduction in maximum and mean dose to LAD in HTp was the only parameter that was statistically significant (LAD-D_max_: χ^2^ = 12.25, *p* = 0.002 and LAD-D_mean:_ χ^2^ = 12.30, *p* = 0.002). In spite of this, using PTVp as target structure increased the mean dose and the volume receiving 5 Gy in the heart. While increasing V5 for heart was statistically significant in VMATp plans (HT vs VMATp, 21.2 ± 9.8 vs 42.7 ± 24.8, *p* = 0.004), it was not significant in HTp plans. The increase in mean heart dose also was found insignificant in both plans.

Moreover, IL-V5 and CL-V5 values were higher for both treatment plans optimized for CTVp when compared to plans based on CTV. While the volume differences were statistically significant in VMATp for both IL and CL (*p* < 0.001 for both), only IL was significant for HTp (*p* = 0.023). The V5 values for both IL and CL were significantly higher in the VMATp when compared to HTp (*p* < 0.001, p = 0.007 respectively). Although IL-V20 was lower for HTp when compared to HT, it was higher for VMATp (*p* = 0.002, *p* < 0.001, respectively).

There was no major change in maximum implant dose since some part of it was still within the PTVp (53.48 Gy for HT, 53,79 Gy for HTp and 53,68 Gy for VMATp, *p* > 0.05). On the contrary, the mean implant doses were dramatically lower in both HTp and VMATp plans as expected (χ^2^ = 27.13, *p* < 0.001). The VMATp technique provided better sparing of the implant with regards to Dmean when compared to HTp technique. (*p*:0.005).

## Discussion

EACG developed a clinical target volume delineation consensus guideline in the setting of IBR with the aim to exclude the implant volume to reduce RT related complications. To our knowledge, this is the first study comparing the dosimetric outcomes of HT and VMAT treatment plans generated based on the EACG clinical target volume delineation. We evaluated OAR and implant doses for retropectorally placed implants, group A, as mentioned in EACG. For this purpose, HT plans based on traditional CTV and both VMATp and HTp plans targeting the CTVp delineated according to EACG were compared. In addition, VMATp and HTp techniques were compared in terms of PTVp coverage, which has a more complex shape compared to traditional PTV.

### Target coverage

We compared VMATp and HTp techniques to determine whichever provided better coverage for the PTVp, since this concave and complex structure presents a challenge for RT planning. Our results showed that both techniques met planning objectives for PTVp coverage with similar homogeneity value. However VMATp plans provided better conformity in comparison to HTp plans.

Although there is abundance of literature evaluating the different techniques for breast radiotherapy, a limited number of studies evaluated the dosimetric data of implant sparing contour-based treatment planning techniques for IBR cases. But, these studies cannot be compared easily to our study because these dosimetric studies have different target volume delineation and dose-fractionation strategies.

Chang et al. compared the dosimetric characteristics of VMAT plans generated based on conventionally delineated clinical target volumes and volumes contoured according to ESTRO ACROP guidelines. Fifteen left-sided PMRT with IBR patients were included in this study and mammaria interna lymph nodes were covered within the target volume [22]. Hypofractionated regimen was used to a total dose of 40.05 Gy in 15 fractions. Given the different fractionation, it is not possible to do a direct comparison with our study, however V95 (%94.3 ± 3.9) is comparable to VMAT plans in our study. Because of the different formalizations were used, the given HI and CI parameters in Chang’s study could not to be compared directly with our resutls.

Massabeau et al. compared 3D-FinF and HT plans for ten breast cancer patients with retropectoral implants [23]. They used similar “pre-implant” target volume PTVp that included the skin, the subcutaneous tissue, the pectoralis muscle and peripheral lymph nodes. Unlike our study, they included internal mammarian lymph nodes to the PTVp. The dosimetric results of our study on PTVp coverage were found to be comparable to the dosimetric results of HT plans in Massabeau et al. study, except for PTVp-V95 metric that showed slightly lower coverage as a result of different objectives for PTVp coverage. Although PTVp presents a challenge for RT planning given the more complex shape when compared with PTV, HI and CN values obtained in our study were found to be comparable with studies evaluating the dosimetrics of whole breast RT.

Haciislamoglu et al.compared five different planning techniques including HT and VMAT for left-sided whole-breast irradiation and their HI values of 0.06 and 0.18 for HT and VMAT, respectively, were comparable to our HI value of HTp and VMATp plans [24].

Therefore in the light of these findings, we can suggest both HT and VMAT techniques can be considered for IBR given the homogeneous and conformal PTVp coverage.

### The heart and LAD

In left-sided cases, although HTp and VMATp lowered Dmax for both LAD and heart, only reduction in LAD Dmax was statistically significant for HTp. HTp and VMATp plans also provided lower LAD Dmean doses when compared to HT, but the differences were only statistically significant for HTp. HTp was significantly better than VMATp for reducing maximum and mean doses to LAD.

Although the exclusion of the implant from the PTVp provided a distance from the heart, surprisingly, mean and V5 doses in the heart increased for both HTp and VMATp plans when compared with HT plans.

Chang et al. presented significantly reduced maximum LAD and mean LAD doses in consistency with our study. In contrast to our study, they also reported lower mean heart doses (3.99 ± 1.02 vs. 5.84 ± 1.78 Gy, p = 0.000), however, these doses were obtained at the cost of higher CB doses. [22].

Massabeau et al. also reported higher heart (7.57 Gy) and LAD (7.15 Gy) mean doses when compared to our HTp plans, most probably secondary to the inclusion of internal mammarian lymph nodes in the target volume in their study [23].

Leonardi et al. aimed to evaluate the dosimetric benefit of an implant sparing approach in patients with IBR undergoing hypofractionated RT (40.05 Gy in 15 fractions) [21]. They compared the dosimetric data of HT plans of 54 conventionally treated IBR patients with 18 IBR patients who were treated with the HALFMOON-CTV technique. Their results suggested that the implant sparing approach provided statistically significant benefit for all OAR doses. When their dosimetric data obtained from the DVH graphs presented in the publication was further evaluated, the HALFMOON technique reduced heart, ipsilateral lung, contralateral lung and contralateral breast volumes within the high dose region while increased within the low dose region, in consistency with our study.

HALFMOON technique suggested by Leonardi et al. failed to reduce the Heart Dmean doses when compared to conventional target based treatment plans. Although the hypofractionated dose scheme led to reduced cumulative heart doses, Dmean heart doses were comparable to our results (4.57 Gy for HTp and 5.65 Gy for VMATp).

### The ipsilateral and contralateral lungs

The V20 is an important metric for IL in breast radiotherapy. Ipsilateral lung volume receiving ≥ 20 Gy (V20) is a predictor of radiation pneumonitis risk, therefore it is aimed to reduce the volume receiving 20 Gy [25–27]. When compared with HT, HTp significantly reduced V20 for IL, while on the contrary VMATp was found to have significantly increased it. Despite higher IL V20 values with VMATp, they were still within our planning limits. The V5 for both IL and CL were higher for both HTp and VMATp techniques when compared to HT, however the increased CL V5 was only significant for VMATp.

Leonardi et al. also showed improvement of IL dose reduction, while HALFMOON technique failed to reduce CL Dmean doses [21].

HTp was found to be superior to VMATp when considering doses to IL and CL.

### The contralateral breast

Radiation induced malignancy is a concern especially for young breast cancer patients [28]. IBR is the treatment of choice especially for younger patients with hereditary gene mutations, therefore secondary breast cancer is an important concern.

HTp techniques succeed in keeping an acceptable range of tolerance for all OAR doses except for CB. When compared to HT plans, CB Dmax and Dmean doses were lower in VMATp plans while they were higher for HTp plans. This difference can be explained by the different arc designs between VMATp and HTp plans.

Lauche et al. aimed to analyse dosimetric results of HT and VMAT techniques in breast and lymph nodes irradiation. They investigated 73 breast patients' plans retrospectively (31 HT and 42 VMAT). Mean CB doses (4.6 ± 0.9 Gy for VMAT, 3.6 ± 0.6 Gy for HT) reported in Lauche O et al. study using simultaneous integrated boost techniques were comparable to VMATp and HT techniques in our study while the doses from our study were higher for HTp [12].

In addition, Chang et al. reported an insignificant increase in Dmax and Dmean for CB. The Dmax and Dmean values for CB were comparable to VMATp results of our study even though the total dose was lower in Chang et al. study.

VMATp provided superior reduction for CB Dmean when compared to HTp. However, this dose reduction in CB was at the expense of increased lower doses (V5) to heart, IL, CL and NT, although they remained within the acceptable range of the tolerance doses for all.

### The normal tissue and liver

Increased low dose radiation to the normal tissue volume is a well known weakness of volumetric arc techniques although it provides better carving of high doses. Despite a smaller target volume, VMATp technique had a higher low dose bath and NT V5 when compared with HT, while the difference between HTp and HT did not reach significance.

Liver V20 values were higher for both VMATp and HTp techniques when compared to HT, but the differences were not significant.

### The implant

Approximately 60% of women undergoing mastectomy for breast cancer undergo IBR, and this practice provides significant psychosocial, cosmetic, and quality-of-life benefits for women [9–11]. Some authors have stated that implant removal is required in more than 31% of patients while 22.3% have major complications secondary to IBR and subsequent PMRT [29–32]. Capsular contracture is the most common complication of breast implants and especially patients undergoing PMRT after IBR are at a higher risk of developing CC. Therefore, limitation of the therapeutic dose to the implant might provide lower risk for CC as well as better cosmesis [12].

Kuske et al. performed brachytherapy on 250 women with implants and limiting the implant dose resulted in lower CC rates of 5% [33]. This data supports the hypothesis that limiting the implant volume receiving the therapeutic RT dose of radiation resulted in reduced CC risk. When the target structure is delineated according to the EACG and a large part of the implant is removed from the target, at least the circumference of this volume can be spared from high doses of radiation. In our study, the planning goals for the implant were Dmean should be less than 38 Gy and the hotspot should not be in it. Although VMATp technique succeeded in keeping the mean implant doses < 38 Gy, HTp failed to meet this constraint to the implant. One can speculate using VMATp may further reduce the rate of CC, which is a major concern after irradiation with the breast implant. However, it should be kept in mind that CC mechanism is not well known and contribution of radiation to the chronic inflammation is only one of the factors including surgical complications, implant material and tumor- tissue microenvironments.

## Conclusion

This study showed that both HTp and VMATp techniques can be considered to deliver homogeneous and conformal doses to CTVp determined according to ESTRO ACROP Consensus Guideline for patients who underwent retropectoral IBR after mastectomy (Group A) while keeping the OAR doses at tolerance levels. While HTp was found to be superior to VMATp for reducing doses to all OAR but CB in comparison to HT, VMATp was found to be superior for providing higher conformity and limiting the implant dose. Given the increased low dose bath volume secondary malignancy risk should be considered, especially for young patients with a long life expectancy.

## Data Availability

The datasets generated during and/or analyzed during the current study are available from the corresponding author on reasonable request.

## Abbreviations

EACG: ESTRO-ACROP Consensus Guideline
CTV: Clinical target volume
CTVp: Clinical target volume based on EACG
PMRT: Post-mastectomy radiation therapy
IBR: Implant-based immediate breast reconstruction
HT: Helical tomotherapy
HTp: HT plan targeted to CTVp
VMAT: Volumetric modulated arc therapy
VMATp: VMAT plan targeted to CTVp
OAR: Organ at risk
NT: Normat tissue
CT: Computerized tomography
RT: Radiotherapy
CC: Capsular contracture
LAD: Left anterior descending artery
IL: Ipsilateral lung
CL: Contralateral lung
CB: Contralateral breast
PTV: Planning target volume
CN: Conformity number
ICRU83-HI: ICRU83 homogeneity index
Vx: Was defined as the percentage of a given tissue volume receiving at least x Gy
Dx%: Was defined as the dose delivered to x% of the volume.
Vpres: Prescribed dose volume (%) inside of the PTV
Avg: Average
STD: Standard deviation
Dmax: Maximum dose
Dmean: Mean dose
3D: Three dimensional
FinF: Field in field
DVH: Dose volume histogram
